# Anasarca in Children

**Published:** 1874-10-15

**Authors:** F. K. Bailey

**Affiliations:** Knoxville, Tenn.


### MEDICAL EXAMINER.

A

*Semi-Monthly Journal of Medical Sciences.*

EDITED BY N. S. DAVIS, M.D., AND F. H. DAVIS, M.D.

No. XX. CHICAGO, OCT. 15, 1874. VOL. XV.

### Original Communications.

### ANASARCA IN CHILDREN.

BY F. K. BAILEY, M.D., KNOXVILLE, TENN.

CASE I. During the spring of 1874 I was called to visit a child, of negro parentage, aged about five. Had measles in winter and when convalescent began to bloat over the whole body. I prescribed a mild alterative to regulate the bowels, to be followed with chlorate of potash and tincture muriate of iron. In a month, or less, the dropsy had disappeared, and the child now (July 15th) is well.

CASE II. Was called July 9th, 1874, to see a child, male, five and one-half years old. The mother is black but the boy has light curly hair, blue eyes, and a dirty yellowish skin.

Was told that the child had not been well for two months or more, but began to swell about the middle of June last. The lower limbs are much swollen; the abdomen full, and the face puffed. Bowels not constipated. Urine red but not very scanty Prescribed chlorate of potash with tincture muriate of iron.

12th. The dropsy is much lessened in the face and abdomen, but still remains in the legs and feet. Scrotum full of water, and the prepuce œdematous. Urine more free. Bowels move freely. Stools watery.

To suspend the chlorate potash and iron. To take iodide potassii in 2 gr. doses three times daily.

15th. Still improving, except that the scrotum is becoming fuller. An eczematous redness upon the scrotum and the thighs, upon which the disturbed parts press. Prescribed a lotion of carbolic acid and hyposulphite soda, to apply, and to powder with fine starch.

I attempted to evacuate the serum from the scrotum, but found the effusion confined to the cellular tissue.

Examined the urine and found an acid reaction, with no cloudiness on applying nitric acid or heat.

Bowels move often; discharges thin, but not unnatural in color. Gave 3 gr. calomel and to continue iodide potassii.

16th. Much the same. The water has escaped from the scrotum and the œdema is less in the legs and feet. Bowels moved freely from the calomel. Passes a good deal of urine. Very thirsty with but little appetite.

18th, 7 P. M. Appears much better. The scrotum less distended, but there is much soreness and redness on its surface as well as on the thighs where the parts touch each other.

To take ½ gr. sulph. cinchonæ every six hours, and continue the iodide potassii.

19th. Urine free, still acid, with no appearance of albumen.

Continue treatment.

21st. General appearances more favorable. Effusion confined to the legs and scrotum. The latter is less distended and not so red.

Have used a lotion of acetate of lead and morphine for two days, which appears to alleviate pain and soreness in the parts. Is taking sulph. cinchonæ, bismuth and salacin, every six hours, and to continue iodide potassii mixture.

26th. Has improved of late. The effusion in the scrotum very much lessened, and he has walked about the house. To-day complains of pain in the stomach. Gave small doses of calomel with bismuth.

28th. Much better. To continue the powders. The mixture to be suspended.

Aug. 3d. Since last report the effusion has all subsided except in the feet. The scrotum completely empty and the abdomen flat. The bowels are still very loose and food is rejected soon after it is swallowed.

Pulse very feeble and slow. Respiration normal. Inclined to sleep more than in former days of its illness.

Directed a resumption of the tincture muriate iron and chlorate potash.

10th. The child becoming more feeble. No effusion even in the feet. Vomiting continues, and the bowels loose.

13th. Failing in strength from day to day.

20th. The child has not vomited for three days and has some desire for food Very much emaciated, but the pulse is slow and the eyes have not lost their lustre.

Some diarrhœa, but not any more profuse than in days past. Gave him small powders of salacin and Dover's powder.

To have what food he will take, to consist of soups and other nutrient articles.

22d. The child appears better, having a good appetite, and the stools of a natural color and consistence.

To continue powders with good food.

28th. This morning no material change can be discovered.

During the past week there has been some irritability of the bowels and more frequent stools, with vomiting during a part of one day. Taken but little medicine, as tonics appear to render the appetite more craving. To have food in moderate quantities. As sleep has been more undisturbed of late, not even a Dover's powder has been required.

Sept. 2d. Found the little fellow more bright this morning and gaining slightly in ability to raise his head. Appetite good and no vomiting. Alvine evacuations frequent but natural in appearance.

Sleeps very well and seems laboring under extreme debility. It now appears to be a mere question of endurance in considering the matter of prognosis.

Sept. 19th. This child continued to linger till about the 12th, when it slowly sank. It is rather unusual to see a case of anasarca continue long enough to admit of the absorption of the effused fluid. In other words, general dropsy proves fatal during its progress, and before any change occurs which will render absorption possible.

I think this case shows, to a remarkable degree, the tenacity of life which is sometimes observed.

There appeared to be no organic disease and death was the result of pure debility.

The above are typical cases of what we often encounter among the colored children. I took no notes in other cases met with during the last six months, but dropsy has been more common than usual during the year. As a result of measles quite a number of cases occurred during the winter and spring. Alterative doses of calomel, followed with tincture muriate of iron and chlorate of potash, was my most frequent tonic.

Case II, above related, was the only one that proved fatal. It is more than probable that the extensive admixture of Anglo-Saxon blood, rendered the child less able to endure the disease. I still adhere to the position that the bleaching process tends to lessen vitality, and, consequently, a mulatto is less able to stand any disease which by its severity makes an inroad upon the constitution.

Besides, quite a proportion of the colored children born since 1865 are of uncertain paternity. The transition of the negro from slavery to freedom has not resulted in any great improvement in moral conduct, and liberty, in too many cases, has lapsed into license, or freedom abused.

Sept., 1874.

